# Long-Range Structures of Amorphous Solid Water

**DOI:** 10.1021/acs.jpcb.1c06899

**Published:** 2021-11-30

**Authors:** Hailong Li, Aigerim Karina, Marjorie Ladd-Parada, Alexander Späh, Fivos Perakis, Chris Benmore, Katrin Amann-Winkel

**Affiliations:** † Department of Physics, AlbaNova University Center, Stockholm University, Stockholm SE-10691, Sweden; ‡ X-ray Science Division, Advanced Photon Source, Argonne National Laboratory, Argonne, Illinois 60439, United States

## Abstract

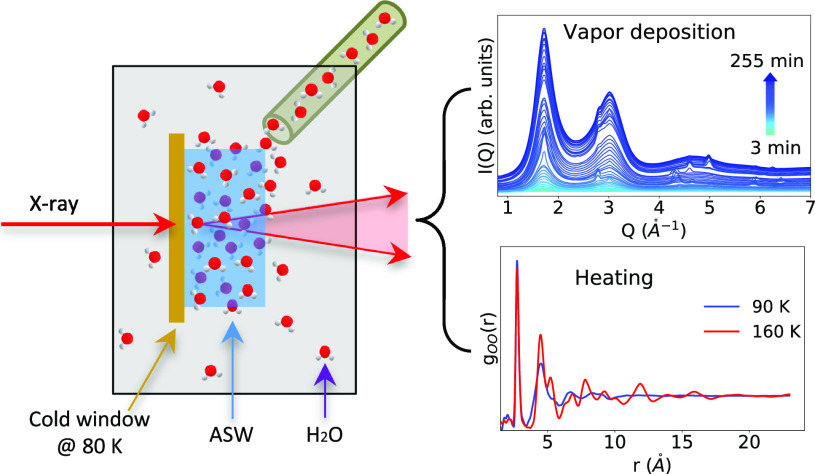

High-energy X-ray diffraction (XRD) and Fourier transform infrared spectroscopy (FTIR) of amorphous solid water (ASW) were studied during vapor deposition and the heating process. From the diffraction patterns, the oxygen–oxygen pair distribution functions (PDFs) were calculated up to the eighth coordination shell and an *r* = 23 *Å*. The PDF of ASW obtained both during vapor deposition at 80 K as well as the subsequent heating are consistent with that of low-density amorphous ice. The formation and temperature-induced collapse of micropores were observed in the XRD data and in the FTIR measurements, more specifically, in the OH stretch and the dangling mode. Above 140 K, ASW crystallizes into a stacking disordered ice, I_sd_. It is observed that the fourth, fifth, and sixth peaks in the PDF, corresponding to structural arrangements between 8 and 12 Å, are the most sensitive to the onset of crystallization.

## 1 Introduction

Amorphous solid water (ASW) is the most abundant form of solid water in astrophysical environments. It is believed to be a major component of interstellar clouds, comets, and solar-system bodies.^1−4^ Due to its porosity and capability of adsorbing gases, ASW is discussed to participate as a medium in many chemical reactions in outer space that could play a key role in the earliest stages of planet building.^3^ However, its true degree of porosity remains unclear due to the lack of unambiguous observational data and a variety of contributing processes during its formation and re-accretion in outer space. ASW was first produced in the laboratory in 1935 by Burton and Oliver through water vapor deposition on a cold copper substrate.^5,6^ The so-formed ASW can serve as a model system to better understand part of the contributing processes. Since then, ASW has been intensively studied.^7−20^ The morphologies and physical properties of ASW fabricated in the laboratory can vary with vapor flux and directionality, substrate temperature, and water partial pressure.^15,21−23^ The effect of irradiation on the structure of amorphous ice, and in particular on the dangling OH groups, has been studied using UV,^24^ IR,^17,25^ and heavy ion bombardment.^26−28^ Formation of high-density ASW has been reported to occur at very low deposition temperatures (<30 K).^29−31^ When the deposition temperature is increased from 77 to 200 K, the deposited ice phase changes from porous ASW (p-ASW)^1^ to collapsed ASW (c-ASW) and eventually to crystalline ice. We note that all of these phases exhibit a low density of around 0.94 g·cm^–3^.^32,33^ The heating process of p-ASW, initially formed at 77 K or below, is accompanied by a pore collapse, hence transforming from p-ASW to c-ASW at temperatures above 120 K.^11,21,34,35^

ASW is known to be structurally similar to low-density amorphous ice (LDA) and hyper-quenched glassy water (HGW). All three types of solid water have a first diffraction peak at 1.7 Å^–1^ in neutron scattering^36^ and present a glass transition at around 136 K.^37^ However, it is not yet fully understood how the p-ASW builds up during vapor deposition nor how the long-range molecular structure changes during the pore collapse.

X-ray diffraction (XRD) and neutron scattering techniques are powerful and non-destructive methods to study molecular-level structures of different materials. They have been previously applied to obtain the structure factors and the pair distribution functions (PDFs) of low- and high-density amorphous ices (LDA and HDA).^37−40^ As ASW is of high astrophysical relevance, most studies investigate the vibrational states *via* Fourier transform infrared spectroscopy (FTIR), while X-ray and neutron scattering studies are, so far, limited.^11,30,34,36^ Taking advantage of the high flux of the high-energy X-rays at beamline 6 ID-D of the Advance Photon Source (APS), we were able to perform *in situ* X-ray measurements on p-ASW during vapor deposition.

The current work presents a combined study of *in situ* XRD and FTIR on ASW, investigating its formation and the pore collapse during heating. Oxygen–oxygen PDFs of ASW were determined, as a function of temperature, up to 23 *Å*. The PDFs were then compared with other amorphous ice forms to investigate potential similarities in the long-range ordering. The OH-stretch vibration mode from the FTIR spectrum was recorded as a function of deposition time and temperature, allowing us to follow the changes in the vibrational spectrum.

## 2 Experimental Methods

### 2.1 Sample Preparation

p-ASW samples for both XRD and FTIR measurements were prepared by vapor deposition of water molecules onto a cold surface. We used ultrapure Milli-Q water with 100% H_2_O composition for the XRD measurements and an isotope diluted sample for the FTIR measurements, with 5 wt % HOD in H_2_O. Deuterium oxide (D_2_O, 99.9 atom % D) was purchased from Sigma-Aldrich. For the FTIR measurements, the sample was additionally degassed by one freeze-pump-thaw cycle.

A liquid N_2_-flow cryostat (JANIS VPF-100, Janis Research Inc.) was used as a sample environment, which is capable of operating in vacuum between 77 K and room temperature. Optical windows allowing for X-ray transmission (Kapton 75 μm and Diamond 50 μm) or IR transmission (CaF_2_ 1 mm) were used, respectively. Water was filled and sealed in a glass vial prior to deposition. Water vapor is guided into the vacuum chamber using a steel tube (1/16″) controlled by a needle valve. The steel tube is directed toward the window at around 45°, 10 mm away from the window. Once a vacuum of at least 10^–3^ mbar and a temperature of 80 K were reached, vapor deposition started by opening the valve. Thereby, p-ASW formed on the cold window surface and grew continuously over time. Co-deposition of impurities due to the relatively high base pressure (10^–3^ mbar), the usage of non-degassed water, and the use of Kapton windows at the cryostat for the X-ray measurement cannot be excluded but are not expected to have an impact on the XRD measurement.

The temperature *T*_m_ was measured by a Si diode. For heating, a resistive heater cartridge (50 Ω) is used. Both are mounted directly at the copper mount of the cold finger. The temperature is controlled by a PID controller (LakeShore 335). However, the Si diode is placed almost 2 cm away from the window itself. According to our previous temperature calibration for the same sample holder, using a powered ice sample, there is an offset of 10 ± 2 K between the sample temperature (*T*_s_) and the measured temperature (*T*_m_), i.e., *T*_s_ ≈ *T*_m_ + 10 K (see the Supporting Information of ref (41)) when using Diamond or Kapton windows. This is also confirmed by FTIR measurements on p-ASW deposited on a Kapton window (see the Supporting Information and Figure S1). Additional FTIR measurements were done using a 1 mm-thick CaF_2_ window that assured a better thermal contact between the sample holder and the window. We assume the *T*_s_ at the CaF_2_ window to be almost the same as *T*_m_ due to a good thermal contact and a value within the experimental error of ±2 K.

### 2.2 High-Energy X-ray Diffraction (XRD)

To investigate the structural evolution of ASW during the vapor deposition and heating processes, *in situ* XRD experiments were performed at beamline 6 ID-D of the Advance Photon Source (APS). Water vapor was deposited on Kapton and Diamond windows for approximately 4 and 2 h, respectively. After the vapor deposition, the p-ASW sample was warmed up to 150 K in steps of 2 K with an overall heating rate of around 0.33 K/min for XRD measurements. The heating rate is estimated by taking into account the accumulation time, heating steps, and dark images taken by the detector in between the measurements.

An unfocused X-ray beam with a diameter of 0.5 mm and a photon energy of 100 keV was chosen. Diffraction patterns were recorded on a large 2D detector (Perkin-Elmer XRD1621), with 2048 × 2048 pixels and a pixel size of 200 μm. This allows for a high momentum transfer *Q*_max_ (23 *Å*^–1^). Cerium dioxide was measured for *Q*-calibration. XRD patterns were collected by accumulating up to 180 individual 2D images, each with an exposure time of 1 s. Background scans were acquired for Kapton and Diamond windows, respectively. After dark image subtraction and azimuthal integration, the intensity *I*(*Q*) *vs Q* curves for ASW were obtained. The oxygen–oxygen structure factor S_OO_(*Q*) of ASW and its corresponding pair distribution function g_OO_(*r*) were derived from the measured intensity *I*(*Q*), following the method described in detail in earlier literature studies.^40,42−44^ Briefly, the total structure factor is derived by applying a weighting function after first subtracting the background and the molecular form factor.^40^ The PDFgetX2 software^45^ was used to apply standard corrections for self-absorption, oblique incidence, and detector efficiency. Finally, the oxygen–oxygen structure factor S_OO_(*Q*) was calculated by subtracting the oxygen–hydrogen contribution from the total S(*Q*), taken from isotope-substituted neutron scattering data for liquid water.^46^ A detailed description of the data analysis can be found in our previous publication;^40^ the current data set was taken during the same beam time and analyzed in the same way. The LDA data^42^ shown in Figure 2a were collected under identical conditions.

### 2.3 Fourier Transform Infrared Spectroscopy (FTIR)

**Figure 1:**
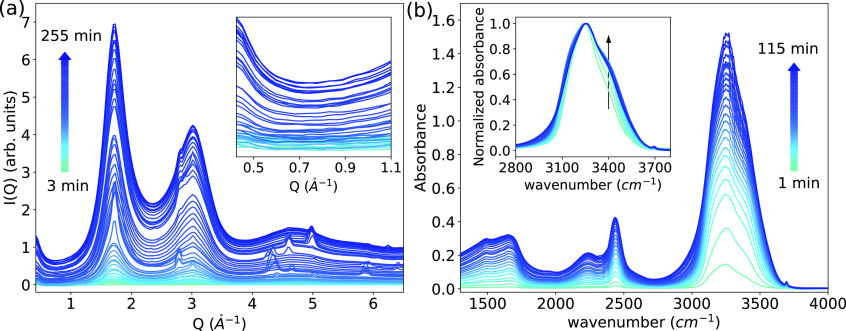
(a) Time evolution of the *I*(*Q*) curves of p-ASW on Kapton during vapor deposition at 80 K. The colored arrow indicates the color code for the deposition time from 0 to 255 min. The inset shows the low *Q* region (0.42 *Å*^–1^ < *Q* < 1.1 *Å*^–1^). (b) Time evolution of the FTIR spectra of p-ASW on CaF_2_ during vapor deposition at 80 K for 115 min. The inset shows the spectra after normalization based on the 3250 cm^–1^ peak.

Similar experiments were performed on an FTIR spectrometer (Frontier, PerkinElmer) using the same sample environment, with both Kapton (see the Supporting Information) and CaF_2_ windows. The FTIR transmission spectra of ASW were measured during vapor deposition for 115 min (Figure 1) and 260 min (see the Supporting Information) and the following heating processes. The p-ASW sample was heated to distinct temperatures between *T*_m_ = 80 and 155 K. The sample was kept at each temperature for 10 min to equilibrate before recording the FTIR spectra. Additionally, we performed a heating-quenching procedure, hence quenching to 80 K in between each heating step, to allow for a same-temperature comparison.

Each spectrum was recorded in a spectral range of 6000–1300 cm^–1^ with a resolution of 2.0 cm^–1^, using an acquisition of six scans with a total duration of 60 s. A background spectrum was taken just before the start of the vapor deposition. Background correction and data acquisition were done using the software “Spectrum 10.5.1” from PerkinElmer.

## 3 Results and Discussion

### 3.1 p-ASW Formation and Growth

Figure 1a shows the development of the intensity *vs* momentum transfer curves, *I*(*Q*), of p-ASW deposited on a Kapton window at 80 K over 255 min. It is observed that two broad diffraction maxima at *Q* = 1.71 and 3.05 *Å*^–1^ appear after the vapor deposition starts and grow continuously with deposition time. These two peaks are characteristic for low-density amorphous ice (LDA) and are correlated to its tetrahedral structure.^42^ The same values were found for p-ASW and LDA samples in earlier work by X-ray and neutron scattering.^36,40^ Thus, we can say that the microstructure of deposited p-ASW is similar to LDA. We also observe an intensity increase along with deposition time (see inset) at small scattering angles (*Q* < 0.7 *Å*^–1^). This is related to p-ASW, as deposited at 80 K, being of heterogeneous nature and forming a porous matrix. In the literature, the degree of porosity was estimated using small-angle neutron scattering (SANS)^11,34^ at around 0.1 *Å*^–1^. However, we were unable to perform such estimations as in the present study we have a limited *Q*-range for we focused on the wide angle, i.e., at *Q* values above 0.4 Å^–1^.

Figure 1b shows the FTIR spectra of p-ASW deposited on a CaF_2_ window at 80 K under similar conditions, recorded in a range of 1300–4000 cm^–1^. The growth of p-ASW is observed from the intensity increase of the main peak located at ν_3_ = 3250 cm^–1^, assigned to OH-stretching vibrations. Similar results were also observed for p-ASW deposited on Kapton and Diamond windows at 80 K (Figures S1 and S2). These vibrations consist of intermolecularly coupled OH oscillators and are related to transverse optical vibration modes (TO).^4,47−51^ The position and line shape of the here reported spectra are consistent with literature values,^4,47,48,51^ taking into consideration that the exact position depends on the deposition rate and angle.^4,15^ The data presented in Figure 1b were recorded for a deposition time of 2 h, instead of 4 h, to avoid saturation of the OH-stretch peak. From the corresponding absorbance of the OH-stretching vibration at 3250 cm^–1^ during the 2 h vapor deposition (Figure S3a), it was observed that the absorbance increases almost linearly after the first 10 min. We determined the thickness of the ASW film as shown in Figure S3b to increase with a rate of around 0.016 μm/min, resulting in a total thickness of 3 μm. To provide a direct comparison to the X-ray data, the spectra for a 4 h deposition are shown in Figure S4. It is observed that the absorbance of the OH-stretching vibration starts to saturate after depositing for more than 2 h.

The normalized data (inset in Figure 1b) show that with increasing thickness of the p-ASW layer, the shoulder at 3400 cm^–1^ also grows. This is also visible when normalizing the data to the OD band (see Figure S3c), as the ratio between the main peak and the shoulder changes continuously. This band has been related to different molecular motions. It was identified as a combination band by Hardin and Harvey,^47^ and later related to longitudinal vibrations (LO) by comparison to the TO–LO splitting in crystalline ices.^48^ It has also been suggested, that the line shape of the OH-stretch region is caused by intermolecular vibrational coupling.^49^ In addition, the surface modes at 3549 and 3503 cm^–1^ are assumed to give a broadening effect to the blue-shifted side of the OH-stretch signal.^25^ Finally, we conclude from our experiments that the observed increase of the shoulder at 3400 cm^–1^ is also indicative of the growing porosity of the p-ASW sample during the deposition process, as this effect happens prior to the saturation of the main peak (compare to Figure S4). Besides the shoulder at 3400 cm^–1^, during deposition, we also observe the appearance of the well-studied three-coordinated dangling bond at 3698 cm^–1^ (non-hydrogen bonded),^25^ which is characteristic for p-ASW.^15^ As such, an increase of the high-frequency shoulder at 3400 cm^–1^ might be related to the appearance of weaker hydrogen bonds, i.e., less water molecules are found to be arranged in a rigid tetrahedral structure. This interpretation is consistent with the temperature dependence of the IR and Raman spectra of liquid water, which exhibit a decrease of the high-frequency spectral component upon cooling.^52^

By using a solution of 5 wt % HOD in H_2_O, another peak at 2440 cm^–1^ was observed and assigned to the uncoupled OD-stretch vibration.^48−51^ At around 1600 cm^–1^, the H–O–H bending mode appears,^53,54^ while the relatively weak peak at around 2230 cm^–1^ has been assigned to a bending + vibration combination band.^55^

### 3.2 Structure of P-ASW

**Figure 2:**
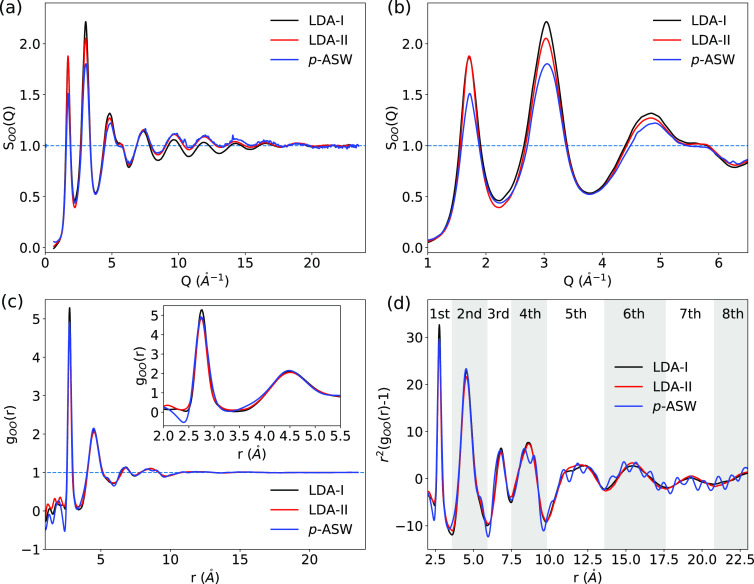
(a) Full-range structure factor, S_OO_(*Q*), at 80 K for p-ASW deposited on Kapton at 80 K for 4 h. LDA-I and LDA-II (Mariedahl *et al*.)^42^ are shown for comparison; both samples were measured under the same conditions. (b) S_OO_(*Q*) curves at a short Q-range. (c) Corresponding pair distribution function, g_OO_(*r*). The inset in (c) shows the short-range correlations. (d) *r*^2^(g_OO_(*r*)-1) curves to help visualize the eight coordination shells up to 23 *Å*.

The structure factor, S_OO_(*Q*), of p-ASW after 255 min is shown in Figure 2a,b (blue line). It is compared with two different LDA samples, namely, the so-called LDA-I (black line) as derived from unannealed high-density amorphous ice (HDA) by heating above its phase transition temperature and LDA-II (red line), prepared through decompression of very high-density amorphous ice at 140 K.^42,56^ The hydrogen bond networks of LDA-I and LDA-II were found to deviate at intermediate length scales.^56,57^Figure 2a shows S_OO_(*Q*) at 80 K for p-ASW, LDA-I, and LDA-II over the full momentum transfer range, up to *Q* = 23 *Å*^–1^. The similarity of the overall oscillations of the three S_OO_(*Q*) curves suggests that the structure of the deposited p-ASW sample resembles that of the LDA structure. However, the intensities for the first three diffraction maxima (Figure 2b) are slightly different for the three differently prepared LDA samples. In particular, the height of the first two maxima for p-ASW is lower than those for LDA-I and LDA-II. Note that the minor peaks at around 11 and 14 *Å*^–1^ are due to the low signal-to-noise ratio of the thin ASW layer that is approximately 5 μm thick (Figure S3b). A direct comparison to eHDA can be found in Figure S5, demonstrating that p-ASW is clearly distinct from high-density amorphous ice.

Figure 2c shows the corresponding oxygen–oxygen pair distribution function, g_OO_(*r*), obtained through Fourier transformation (FT). The difference in S_OO_(*Q*) is observed as a small difference in g_OO_(*r*) in the second coordination shell. The inset in Figure 2c shows g_OO_(*r*) values of the first and second coordination shells at 2.74 and 4.5 *Å*, respectively. It is well known that the second coordination shell is connected to the tetrahedrality of the hydrogen-bond network structure.^40,58^ For p-ASW (blue curve), we observed a slightly higher g_OO_(*r*) value at around 3.7 *Å*, compared to LDA. The difference in S_OO_(*Q*) and the enhancement in g_OO_(*r*) at 3.7 Å are caused by the microporous nature of the p-ASW sample and relates to a lower degree of tetrahedrality for p-ASW. The second peak in the g_OO_(*r*) of HDA is also located at this value and assigned to the appearance of interstitial, non-tetrahedrally hydrogen-bonded molecules.^40^ To emphasize the coordination shells up to 23 *Å*, *r*^2^(g_OO_(*r*)-1) is shown in Figure 2d.

We were able to resolve up to eight coordination shells due to the wide *Q*-range accessed in the measurement. The small oscillations that are visible in the *r*^2^(g_OO_(*r*)-1) curve for p-ASW are caused by the lower signal/noise ratio in the micrometer-thick ice film. Still, we consider the overall structure of p-ASW to be very similar compared to LDA-I and LDA-II, as all coordination shells are found to be located at similar distances. Therefore, we can conclude that the molecular structure of the vapor-deposited p-ASW sample deposited at 80 K exists within the same energy landscape megabasin of LDA. Our results are consistent with previous findings,^40,42,56^ and additionally extend the previously explored coordination shells up to a distance of 23 Å.

### 3.3 Structural Evolution of ASW during the Heating Process

**Figure 3:**
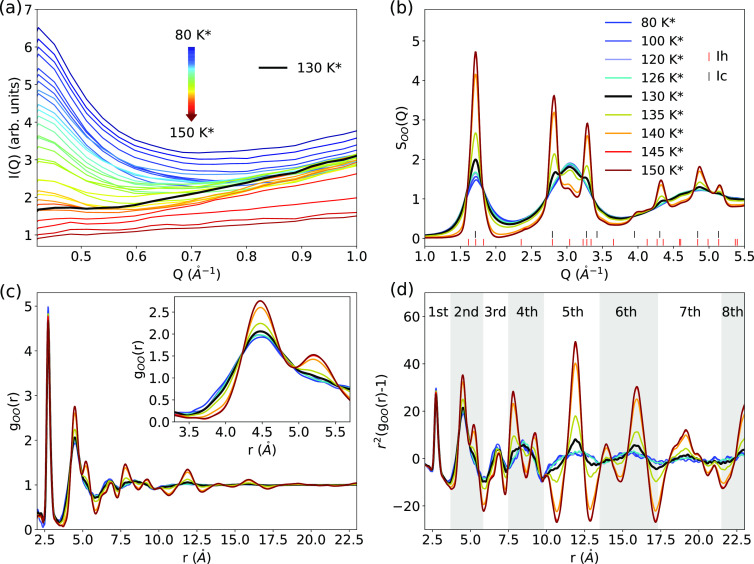
(a) *I*(*Q*) curves in the small *Q* region (SAXS) of selected temperatures of ASW deposited on Kapton at 80 K for 255 min. The arrow indicates the color code for *T*_m_ from 80 to 150 K. (b) Representative structure factor, S_OO_(*Q*), for ASW (same as in (a)) during heating. The corresponding g_OO_(*r*) and *r*^2^(g_OO_(*r*)-1) for ASW are plotted in (c) and (d), respectively. The inset in (c) shows the short-range correlations. *Note: the temperature was measured at the cold finger (*T*_m_); we assume the sample temperature (*T*_s_) to be warmer by 10 ± 2 K (*T*_s_ ≈ *T*_m_ + 10 K).

To investigate the crystallization behavior of p-ASW, we followed its diffraction patterns during heating in steps of 2 K. Figure 3a shows the *I*(*Q*) curves of selected temperatures, in the small *Q* region (0.42 *Å*^–1^ < *Q* < 1.0 *Å*^–1^), of p-ASW when heating it up to *T*_m_ = 150 K (assumed that *T*_s_ = 160 K). Opposite to the trend observed in the inset of Figure 1a, the intensity of the *I*(*Q*) curves decreases continuously with increasing temperature up to *T*_m_ = 130 K (assumed that *T*_s_ = 140 K, black curve). This intensity decrease is most obvious at *Q* < 0.7 *Å*^–1^, indicating that the formed micropores (p-ASW) steadily collapse (c-ASW) when heating up to *T*_m_ = 130 K. Above 130 K, the intensity at *Q* < 0.7 *Å*^–1^ decreases less with increasing temperature, meaning that most of the pores collapsed during the previous heating process (80 K < *T*_m_ < 130 K).

The structure factor S_OO_(*Q*) of ASW at representative temperatures is plotted in Figure 3b. The positions of the Bragg peaks for hexagonal ice (ice Ih)^59^ and cubic ice (ice Ic)^60^ are marked in Figure 3b as red and black vertical lines, respectively. Although the micropore collapse is observed at temperatures below *T*_m_ = 130 K (Figure 3a), no pronounced structural change is seen on the molecular level, evidenced by the almost identical S_OO_(*Q*) curves (Figure 3b). Crystallization is observed at temperatures above *T*_m_ = 130 K (*T*_s_ = 140 K) by the appearance of Bragg peaks at *Q* = 2.79 and 3.28 *Å*^–1^, both of which are related to ice Ic and ice Ih.^42^ Some Bragg peaks strictly related to ice Ih, *e.g*., *Q* = 3.04 *Å*^–1^, are observed at higher temperatures. Thus, we conclude that ASW transforms from LDA to the so-called stacking disordered ice I (ice I_sd_) at around *T*_s_ = 140 K.^60−66^ A direct comparison of crystalline ice derived from p-ASW (warmed to *T*_s_ = 160 K), LDA-I (warmed to *T*_s_ = 150 K), and LDA-II (warmed to *T*_s_ = 160 K) is shown in Figure S6. While the ice Ih peak at *Q* = 3.04 *Å*^–1^ is absent in the crystalline ice derived from LDA-II, it is most pronounced in the LDA-I sample and appears as a minor feature in S_OO_(*Q*) of ice I_sd_ (p-ASW heated to *T*_s_ = 160 K), featuring a mixture of both the S_OO_(*Q*)s for ice Ih and ice Ic.^42,62^ Note that this terminology is used to discriminate the different topological structures of ice I.^60−62,66,67^

The g_OO_(*r*) and *r*^2^(g_OO_(*r*)-1) curves for ASW at the selected temperatures are plotted in Figure 3c,d, respectively. Similar to S_OO_(Q), almost no changes were observed for the g_OO_(*r*) curves at temperatures below *T*_m_ = 130 K. Above *T*_m_ = 130 K, the height of the second coordination shell (*r* = 4.5 *Å*) increases alongside temperature (inset in Figure 3c). This observation can be associated with the ordering of two adjacent tetrahedral structures. Meanwhile, the broad fifth and sixth coordination shells start to sharpen. This trend is consistent with the results from super-cooled water studied by Pathak *et al.*,^68^ where a similar fifth peak of water at *r*≈ 11 *Å* is associated with five-membered pentamer rings in low-density liquid-like structures. Thus, the fifth and sixth coordination shells are sensitive to the long-range order structures. At the same time, a feature at *r* = 5.3 *Å* appears at *T*_m_ > 135 K (*T*_s_ > 145 K), indicating the presence of hexagonal stacking. The previous observation supports the S_OO_(Q) results in Figure 3b, which show that ASW changes from LDA to ice I_sd_.

For all X-ray measurements shown in Figures 2 and 3, water vapor was deposited on Kapton. We also used a Diamond window as an alternative substrate, where ASW was tracked during vapor deposition and heating using X-rays. The corresponding S_OO_(*Q*), g_OO_(*r*), and *r*^2^(g_OO_(*r*)-1) are summarized in Figures S7 and S8. It is observed that deposited p-ASW transformed into ice I_sd_ already during the deposition process (Figure S7a). This quick crystallization might be caused by the lower energy barrier to form a crystalline structure on the diamond surface, which on the other hand, seems unlikely in a micrometer-thick layer of ice. Another cause could be the weak adhesion between ice and diamond, leading to a bad thermal contact. When heating up, the ice I_sd_ restructures and increases the fraction of ice Ih with increasing temperature evidenced by the additional Bragg reflections (Figure S8a).

**Figure 4:**
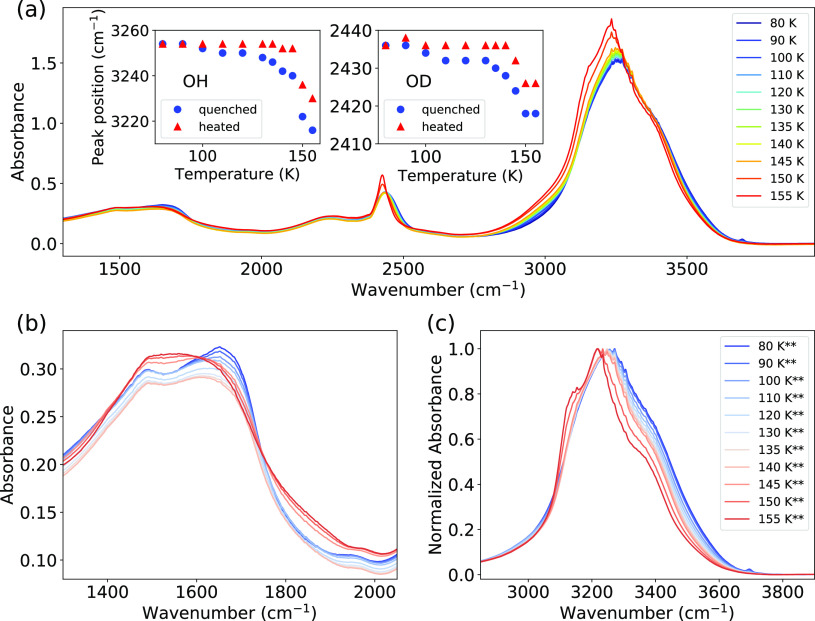
(a) FTIR spectra of ASW during step-wise heating from 80 to 155 K. Inset in (a) presents the peak positions of the OH- and OD-stretch modes during heating and quenching. (b, c) FTIR spectra of OH-bend and OH-stretch modes for ASW, respectively, after quenching^**^ to 80 K from different temperatures. The spectra in (c) is normalized to the main peak. Note that temperature was measured at the cold finger (*T*_m_); we assume the sample temperature (*T*_s_) at the CaF_2_ window to be almost the same (*T*_s_ = *T*_m_) due to a good thermal contact.

In an independent measurement, the FTIR spectra were recorded to track the vibrational changes of the OH and OD stretching regions of ASW during heating. Figure 4a shows the absorption spectra collected at different temperatures during the heating process. With the increasing temperature from 80 to 130 K, it is observed that the FTIR peak for the OH stretch mode at ν_3_ = 3250 cm^–1^ narrows, and the dangling OH group at 3698 cm^–1^ disappears. Hence, the microporous structure for p-ASW collapses during heating, in agreement with the literature studies.^11,15,34,69,70^ In the inset of Figure 4a, the peak positions of OH and OD stretch modes are plotted as a function of temperature. There, we observe a shift toward lower frequency at 150 K due to crystallization. Similar peak shift behavior was reported by Mishima and Suzuki^71^ and Salzmann *et al*.^12^ in their Raman spectroscopy results. Also, the OH bending mode clearly changes with crystallization, as seen in the quench-recovered spectra recorded at 80 K (Figure 4b). While the bending mode of ASW shows two separated peaks, they merge to one broader band at 145 K.^53^Figure 4c shows the normalized absorption spectra of OH stretch modes for the ASW sample after quenching the sample to 80 K from different temperatures. The observed collapse is even more evident after quenching than in the measurements made at high temperatures (Figure 4c).

Above 140 K, the FTIR peaks for both the OD and OH stretch modes are red-shifted, indicating stronger hydrogen bonds, and can be explained by the distortion of the hydrogen bonds in ASW compared to crystalline ice, a phenomenon well discussed in the literature.^47−49^ This trend has been previously observed during crystallization of hyperquenched glassy water when heating from 40 to 150 K at 5 K/min.^69^ In liquid water, a similar behavior has been reported, where a colder sample exhibits a more red-shifted absorption frequency.^19^ Generally, the positions of the OD and OH stretch modes on the FTIR spectrum of ice derived from ASW at 155 K are very similar to the ones for ice Ih,^50^ but their widths are much broader. In addition, we observe that the OH bending mode in the region of 1600 cm^–1^ is split in two peaks in the ASW spectrum at temperatures below 145 K (Figure 4).

## 4 Conclusions

In this study, we performed *in situ* high-energy XRD experiments for ASW during vapor deposition and heating processes, in which the ASW’s long-range structure factor and oxygen–oxygen correlations were determined, up to a distance of *r* = 23 *Å*. We have also recorded, in an independent *in situ* FTIR experiment, the OH and OD stretch vibration modes, using the same sample chamber as in the XRD measurements. We have demonstrated that the combination of *in situ* XRD and FTIR measurements allows for a comprehensive description of the structural evolution of the vapor-deposited p-ASW.

A microporous structure (p-ASW) was observed to build up during the vapor deposition at 80 K over 250 min. The growth of the deposited layer was observed through an increase in the total X-ray intensity (*I*(*Q*)) as well as an increase in absorbance in the OH-stretch vibrational region at around ν_3_ = 3250 cm^–1^. The porous characteristic of the p-ASW sample was observed through an increased SAXS intensity (*Q* < 0.7 *Å*^–1^) in the XRD measurements, as well as the appearance of dangling OH bonds at 3698 cm^–1^. When increasing the temperature, the micropores collapse, which is seen as a decrease of the shoulder at 3400 cm^–1^, vanishing of the dangling OH bond, and a decrease in SAXS intensity. This result is consistent with a previously reported SANS experiment.^11,34^ The pair correlation functions g_OO_(*r*) of p-ASW and LDA are almost identical up to 23 Å (Figure 2c), which is consistent with the fact that both ASW and LDA have the same density.^32^ The main structural difference is seen in the region around 3.7 Å and indicates the existence of interstitial molecules in the porous ASW structure, as tetrahedrally coordinated LDA does not have an enhancement at this distance.^40^ The structure of p-ASW is also clearly distinct from HDA, as can be seen in a direct comparison in Figure S5.

Above a sample temperature of 140 K, we observed that the fourth, fifth, and sixth peaks in the PDF are the most sensitive to the onset of crystallization, indicating the growth of the local tetrahedral network at length scales of 8–16 Å (black curve in Figure 3d). In particular, the growth of the fifth shell at 12 Å is related to the formation of hexagonal rings.^68^ At temperatures above 145 K, the second, fourth, and sixth peaks, they each split into two peaks throughout crystallization (Figure 3d). By comparing with PDFs of crystalline ice derived from different LDA ices, we observe that ASW transforms into ice I_sd_ above 140 K (see the Supporting Information). This study is of relevance for understanding the microstructure of ASW as a major component involved in chemical processes in interstellar ices.

## Supporting Information Available

It contains additional FTIR measurements for vapor deposition using CaF_2_, Kapton, and Diamond windows; the measurements on Kapton serve as temperature calibration for the X-ray data; the structure factor, S_OO_(*Q*), and the corresponding pair distribution function, g_OO_(*r*), for p-ASW deposited on a Diamond window during vapor deposition at 80 K and during the heating processes are shown (PDF)
