# Supplementary material for: Long-Range Structures of Amorphous Solid Water
Source: J Phys Chem B. 2021 Nov 30;125(48):13320–8. doi: 10.1021/acs.jpcb.1c06899 (PMC8667042; doi:10.1021/acs.jpcb.1c06899)
Supplement: Supplementary file 1 — jp1c06899_si_001.pdf [file jp1c06899_si_001.pdf]

Supporting Information:
Long-Range Structures of Amorphous Solid Water

Hailong Li[1], Aigerim Karina[1], Marjorie Ladd-Parada[1], Alexander Späh[1], Fivos Perakis[1], Chris Benmore[2], and Katrin Amann-Winkel[1#*]

[1.] Department of Physics, AlbaNova University Center, Stockholm University, SE-10691 Stockholm, Sweden.

[2.] X-ray Science Division, Advanced Photon Source, Argonne National Laboratory, Argonne, Illinois 60439, United States.

[#] current address: Max Planck Institute for Polymer Research, 55128 Mainz & Institute of Physics, Johannes Gutenberg University Mainz, 55128 Mainz, Germany

* Corresponding author: katrin.amannwinkel@fysik.su.se; amannk@mpip-mainz.mpg.de

Supporting Information

FTIR measurements

Porous amorphous solid water (p-ASW) was prepared via vapor deposition on a cold substrate, using three different types of substrates, namely CaF_2 (1 mm), Kapton (75 μm) and Diamond (50 μm). Kapton and Diamond was chosen for the X-ray measurements and has been used for the FTIR measurements for comparison.

p-ASW vapor deposited on Kapton

As in the X-ray experiments, we also performed FTIR measurements using Kapton as substrate. Figure S1a shows the growth of p-ASW within 120 min, and Figure S1b shows the subsequent heating. As for the X-ray data (Figure 3), crystallization is observed at a cryostat temperature Tm of 130 – 135 K. The crystallization at the CaF_2 window instead takes place at Tm = 145 K. This confirms the estimated temperature offset between cryostat temperature and sample temperature when using a Kapton window, due to bad thermal contact. This is also consistent with our previous calibration on powder samples using the same cryostat (see supplementary material of Perakis et al.[1]). We would like to point out, that the peak at around 3600 cm^{-1} showing up at high temperatures in Figure S1b is not real, and most likely caused by the poor

reference subtraction when using a Kapton window. The Background reference was measured at 80 K.

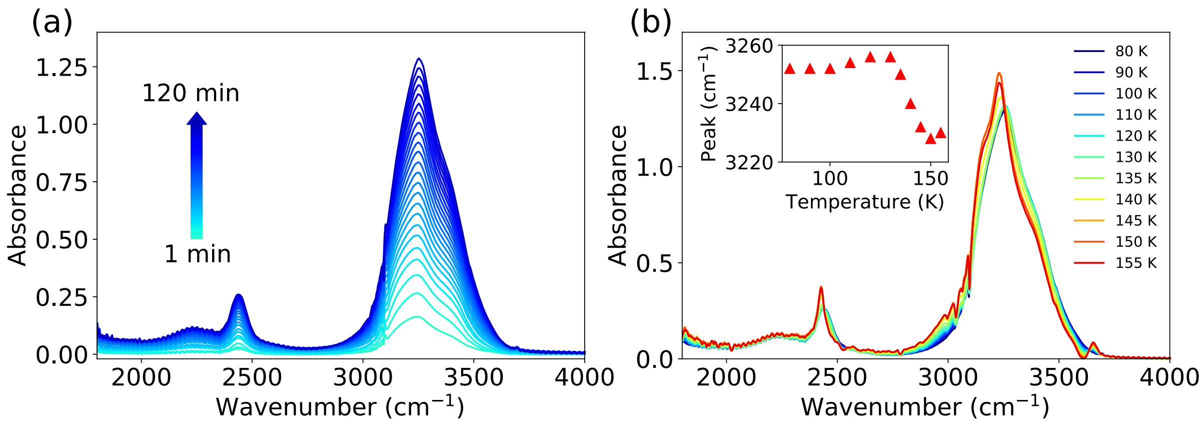

Figure S1. (a) Time evolution of the FTIR spectra of p-ASW on Kapton during vapor deposition at 80 K. (b) FTIR spectra recorded during heating, allowing for 10 min equilibration at each temperature. The inset shows the main peak position as a function of temperature. The temperature was measured at the cold finger, crystallization temperature confirms the sample temperature to be warmer by 10 K ± 2 K when using Kapton.

p-ASW vapor deposited on diamond

To investigate the influence of the cold surface on the deposited p-ASW, X-ray diffraction and FTIR measurements were also performed using a 50 μm thick and 7.8 mm diameter diamond window (optical grade and polished, from Advanced Diamond Technology). The oscillations on the spectra are presumably caused by reflections of the diamond window itself.

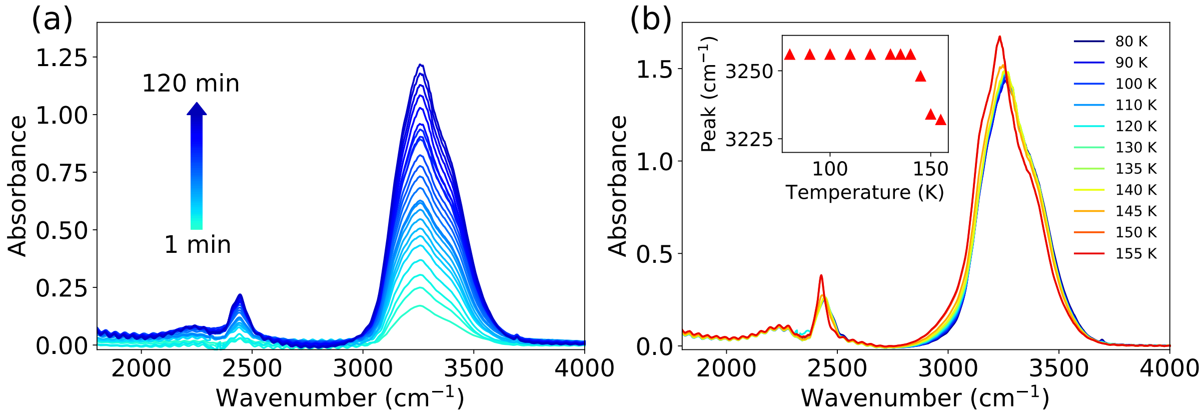

Figure S2. (a) Time evolution of the FTIR spectra of p-ASW on Diamond during vapor deposition at 80 K. (b) FTIR spectra recorded during heating, allowing for 10 min of equilibration at each temperature. The inset shows the main peak position as a function of temperature.

p-ASW vapor deposited on CaF_2

Figure 1b shows p-ASW as deposited on a CaF_2 window for 115 min. The corresponding absorbance of the OH-stretching vibration at 3250 cm $^{-1}$ is plotted in Figure S3a. It is observed that the absorbance increases almost linearly after the first 10 min. This suggests that the FTIR detector is not saturated within 2 hours vapor deposition although the absorbance value is relatively high. The thickness of the ASW film was calculated according to the Beer's law[2]:

$$A_i(\tilde{\nu}) = \frac{1}{\ln 10}\alpha_i(\tilde{\nu})bc_i \qquad \text{(S1)}$$

where $A_i(\tilde{\nu})$ is the absorbance of component i at wavenumber $\tilde{\nu}$, b is the sample thickness, α_i $(\tilde{\nu})$ is the linear absorption coefficient (cm $^{-1}$) of component i at $\tilde{\nu}$, c_i is the concentration of component i. For the OH-stretching vibration at 3250 cm $^{-1}$ of water, the α_{OH} is around 1.25 $\times$ 10^4 cm $^{-1}$ according to the literature.[3] The calculated ASW thickness is presented in Figure S3b. It is observed that the thickness increases much faster in the first 10 min deposition, then it increases linearly with a rate of around 0.016 μm/min, resulting in a total thickness of 3 μm after 2h deposition time.

Alternatively to the normalization to the OH stretch band at 3250 cm $^{-1}$, shown in the inset of Figure 1b, we here normalized the spectra to the OD-stretch band (2440 cm $^{-1}$) instead. This is presented in Figure S3c. The ratio between the main peak at 3250 cm $^{-1}$ and the shoulder at 3400 cm^{-1} changes continuously. This is consistent with our interpretation discussed in the main manuscript that the shoulder at 3400 cm^{-1} is indicative for the growing porosity of the p-ASW sample during the deposition process. In an independent measurement, the vapor deposition on CaF_2 window was monitored for 260 min (Figure S4), similar to the X-ray experiment. Compared to Figure 1b, the OH stretch region is observed to saturate after 2 hours, as seen in the absorbance in Figure S4b. Taking into account the full timescale of 4 h, we observed the absorbance to increase non-linearly. This saturation effect is additionally visible when normalizing the spectra to the OD-stretch band (Figure S4c), as the OH band then first decreases

but increases again. From the overall growth rate of both the OH and the OD band, we estimate a total thickness of around 5 μm after a deposition time of 4 h.

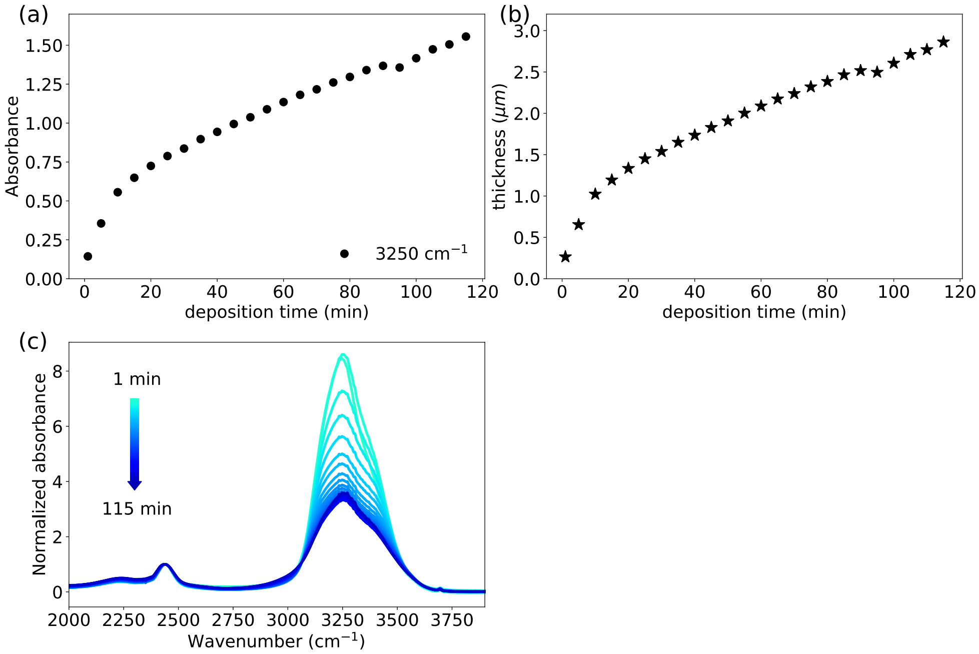

Figure S3. (a) The absorbance of the OH-stretching vibration at 3250 cm $^{-1}$ during 2 hours vapor deposition. It is calculated by using the data in Figure 1b. (b) The corresponding thickness of the ASW film. (c) The spectra after normalization based on the 2440 cm^{-1} peak.

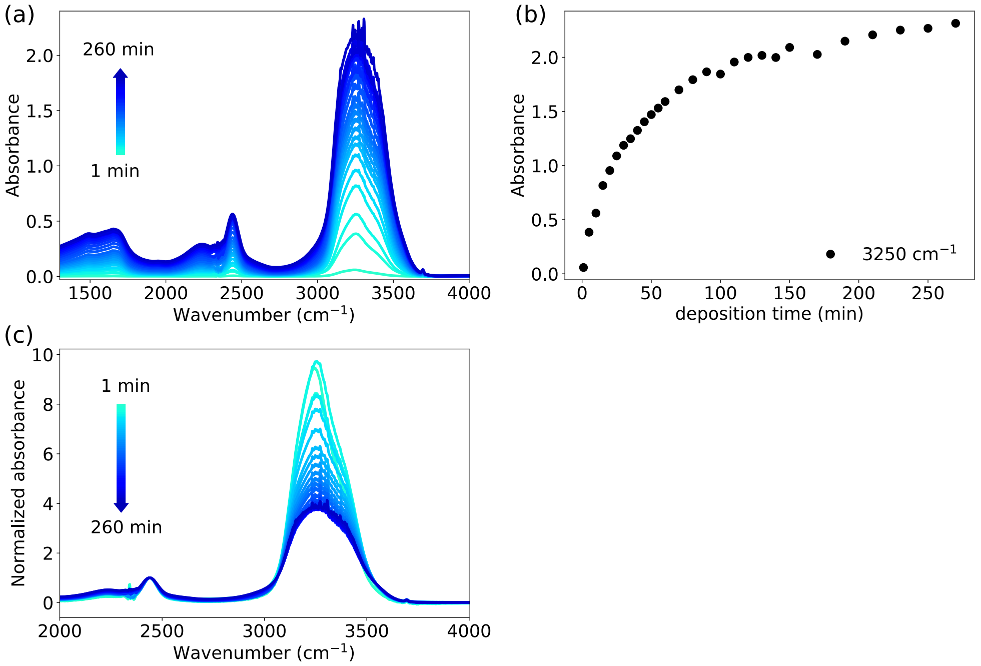

Figure S4. (a) Time evolution of the FTIR spectra of p-ASW on CaF_2 during vapor deposition at 80 K. (b) The corresponding absorbance of the OH-stretching vibration at 3250 cm $^{-1}$ during 4 hours vapor deposition. (c) The spectra after normalization based on the 2440 cm^{-1} peak.

Structure factor of eHDA and p-ASW at 80 K

The structure factor of p-ASW at 80 K is compared with equilibrated high-density amorphous ice (eHDA), which was prepared through decompression of very high-density amorphous ice to 0.07 GPa at 140 K, then quenched to 80 K. The presented eHDA-data is replotted from our previous publication.[4] It has been measured by our group under the same conditions at the same beamline.

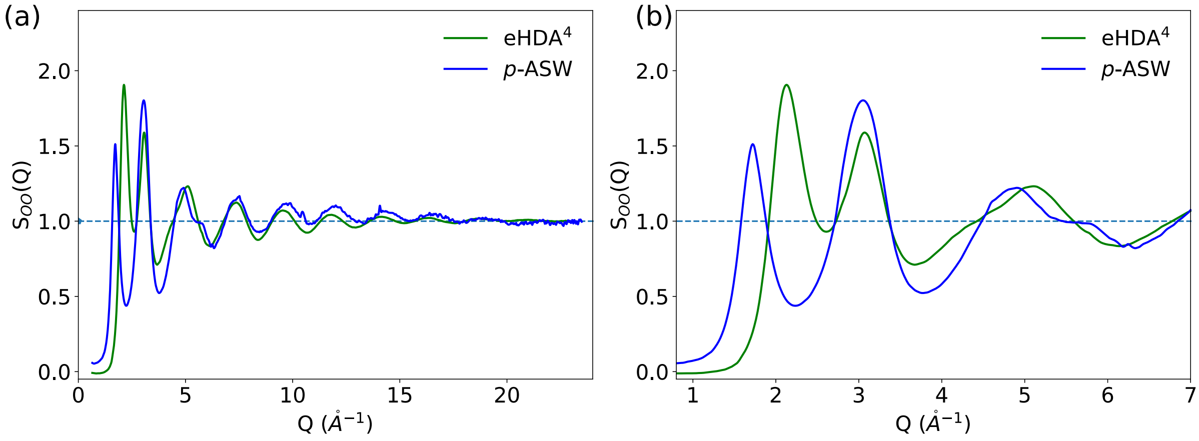

Figure S5. (a) Structure factor, $S_{OO}(Q)$, at 80 K for p-ASW deposited on Kapton at 80 K for 4 hours and eHDA.[4] eHDA-data is taken from literature, but had been measured by our group under the same conditions. (b) $S_{OO}(Q)$ curves at short Q-range.

Crystalline ices derived from warming ASW, uHDA, and eHDA

The structure factor for crystalline ice derived from heating p-ASW to a sample temperature of T_s = 160 K, and crystalline ice derived from different high-density amorphous ices (HDA) was compared. This is, unannealed HDA (uHDA) transforms to LDA-I and crystallizes at Ts = 150 K, while equilibrated HDA (eHDA) transforms to LDA-II and crystallizes at Ts = 160 K. The comparison of the structure factor for these three states is shown in Figure S6. To help assign the crystal structure derived from ASW at temperatures above 140 K, the positions of the Bragg peaks for ice Ih and ice Ic are marked as red and black vertical lines, respectively. From our previous work,[4] we know that LDA-I crystallizes to ice Ih, while LDA-II crystallizes into a more cubic ice Ic, as can be seen by the lack of hexagonal Bragg peaks at Q = 2.36 $Å^{-1}$ and

3.04 Å^{-1}. However, the $S_{OO}(Q)$ curve of ASW at 150 K is located between that of LDA-I and LDA-II. Thus, p-ASW transforms to ice I_{sd}.

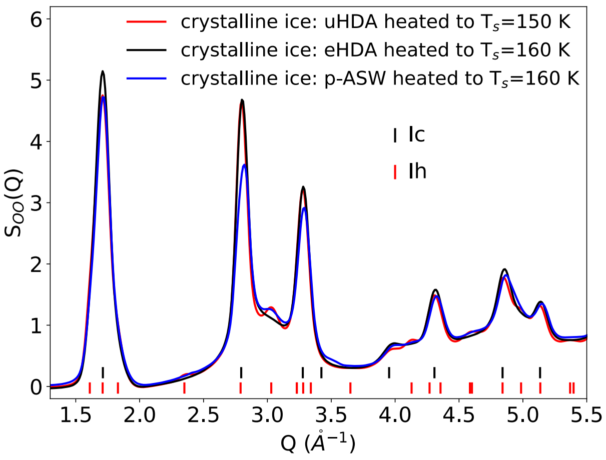

Figure S6. Structure factor $S_{OO}(Q)$ for crystalline ice derived from p-ASW (deposited on Kapton window at 80 K) at T_{sample} = 160 K, crystalline ice derived from uHDA through LDA-I at T_s = 150 K, and crystalline ice derived from eHDA through LDA-II at T_s = 160 K.[4] The red and black vertical lines mark the positions of the ice Ih[5] and ice Ic[6], respectively.

X-ray measurements for vapor deposition on Diamond window

The calculated $S_{OO}(Q)$, $g_{OO}(r)$, and $r^2(g_{OO}(r)$-1) of ASW on Diamond are summarized in Figure S7. Surprisingly, the freshly formed p-ASW begins to crystallize after 30 minutes of vapor deposition, evidenced by the appearance of Bragg peaks at Q = 2.79 Å^{-1} and 3.28 Å^{-1}.[4] This was not observed on the FTIR measurements using the diamond window (Figure S2), and also not seen using Kapton or CaF_2. Therefore, for ASW formed on Kapton, 180 frames have been accumulated, while no change was observed during this total time of 180 s, this increases the signal to noise ratio compared to a single measurement. For ASW formed on diamond, only 60 frames were summed up. The crystalline ice formed during vapor deposition is missing the ice Ih Bragg peaks at Q = 2.36 Å^{-1} and 3.04 Å^{-1}. Therefore, we consider that the p-ASW deposited on diamond crystallizes to ice I_{sd}.[7–11]

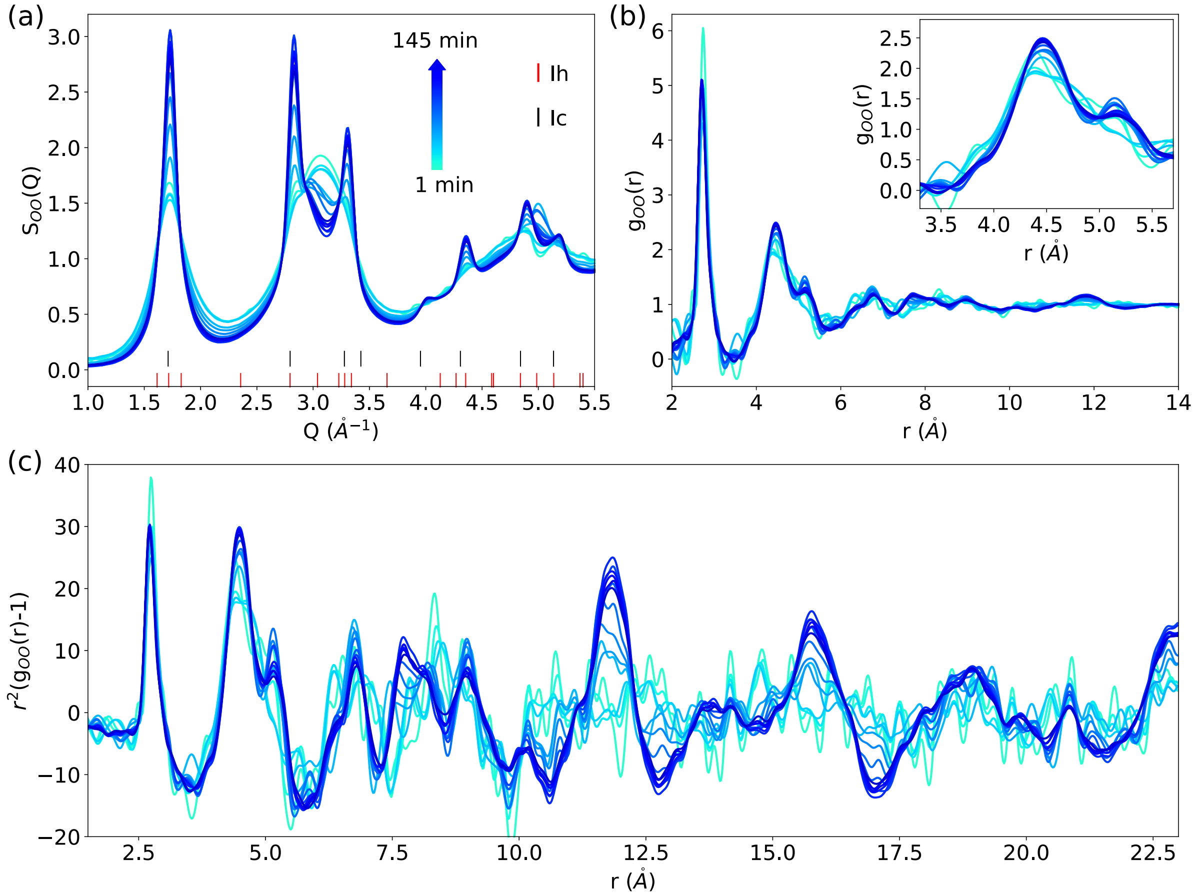

Figure S7. Evolution of the structure factor $S_{OO}(Q)$, $g_{OO}(r)$, and $r^2(g_{OO}(r)$-1) for ASW during vapor deposition on diamond, at 80 K for 145 minutes. The red and black vertical lines in (a) mark the positions of the ice Ih and ice Ic, respectively.

To investigate the stability of the formed ice I_{sd} on the diamond window, it was heated to different temperatures (T_m = 100, 150, 200, and 250 K). The calculated $S_{OO}(Q)$, $g_{OO}(r)$, and $r^2(g_{OO}(r)$-1) are summarized in Figure S8. An additional hexagonal Bragg peak at Q = 2.36 $Å^{-1}$ appears at temperatures above 200 K (Figure S8a). This peak indicates that there is an increase in hexagonal stacking in the crystalline structure.

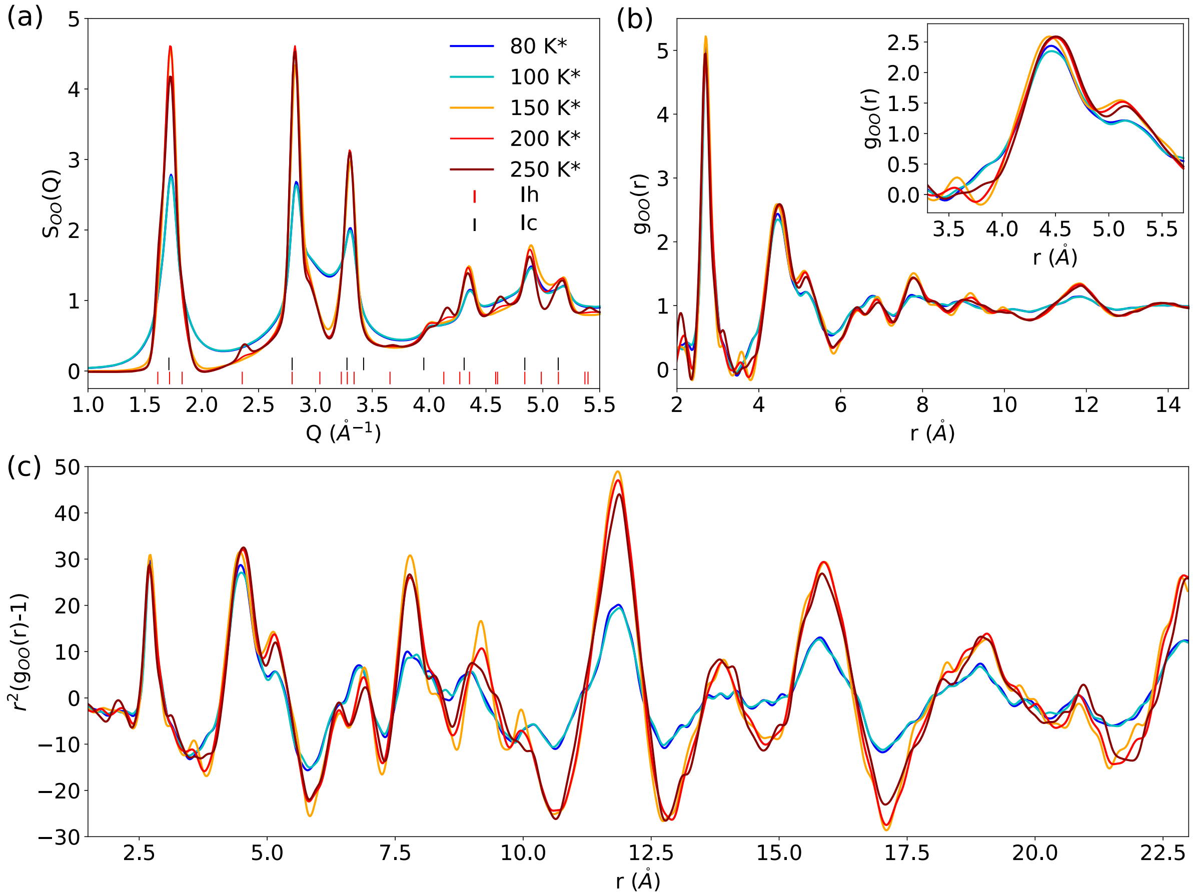

Figure S8. Evolution of the structure factor $S_{OO}(Q)$, $g_{OO}(r)$, and $r^2(g_{OO}(r)-1)$ for ice I_{sd}, derived from ASW which was initially deposited on diamond for 145 minutes, and then heated from 80 K to 250 K. The red and black vertical lines in (a) mark the positions of the ice Ih and ice Ic Bragg peaks, respectively. *Note: the temperature was measured at the cold finger (T_m), we assume the sample temperature (T_s) to be warmer by 10 K ± 2 K ($T_s \approx T_m + 10$ K).

Finally, we would like to add a comparison of our X-ray data with neutron scattering results from Playford et al.[12] The $g_{OO}(r)$ curves for the formed crystalline structure (ice I_{sd}) at T_s = 160 K (green) and 260 K (red) are plotted in Figure S9 in the range of 7-15 Å. They are compared with the differential correlation functions D(r) calculated from neutron data for I_{sd} samples annealed at 156 K (blue) and 201 K (orange).[12] A direct comparison of the different datasets is difficult and might be misleading, as the signal to noise ratio in our experimental X-ray setup as well as the resolution of the detector does not allow a detailed analysis of the crystalline structures. Nevertheless, all four datasets agree well, within the limitations described above, and show similar features at the same length sales.

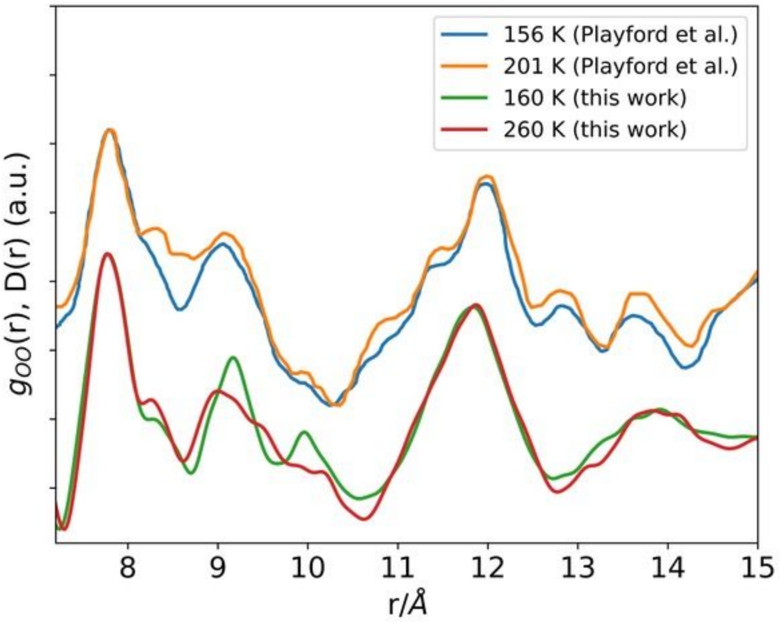

Figure S9. The comparison of $g_{OO}(r)$ curves for I_{sd} sample, which was initially formed on Diamond window at 80 K then heated up to T_s = 160 K and 260 K, and the differential correlation functions D(*r*) calculated from neutron data from Playford et al. for I_{sd} samples annealed at 156 K and 201 K.[12]

References

(1) Perakis, F.; Amann-Winkel, K.; Lehmkühler, F.; Sprung, M.; Mariedahl, D.; Sellberg, J. A.; Pathak, H.; Späh, A.; Cavalca, F.; Schlesinger, D.; et al. Diffusive Dynamics during the High-To-Low Density Transition in Amorphous Ice. *Proc. Natl. Acad. Sci. U. S. A.* **2017**, *114*, 8193–8198.

(2) Griffiths, P. R.; De Haseth, J. A. *Fourier Transform Infrared Spectrometry*; John Wiley & Sons, 2007; Vol. 171.

(3) Elshik, E.; Bester, R.; Nel, A. Appropriate Solar Spectrum Usage: The Novel Design of a Photovoltaic Thermal System. *2016 Ashrae Winter Conf. Pap.* **2016**, 3–9.

(4) Mariedahl, D.; Perakis, F.; Späh, A.; Pathak, H.; Kim, K. H.; Benmore, C.; Nilsson, A.; Amann-Winkel, K. X-Ray Studies of the Transformation from High- To Low-Density Amorphous Water. *Philos. Trans. R. Soc. A Math. Phys. Eng. Sci.* **2019**, *377*, 20180164.

(5) Dowell, L. G.; Rinfret, A. P. Low-Temperature Forms of Ice as Studied by X-Ray Diffraction. *Nature* **1960**, *188*, 1144–1148.

(6) del Rosso, L.; Celli, M.; Grazzi, F.; Catti, M.; Hansen, T. C.; Fortes, A. D.; Ulivi, L. Cubic Ice Ic without Stacking Defects Obtained from Ice XVII. *Nat. Mater.* **2020**, *19*, 663–668.

(7) Kuhs, W. F.; Sippel, C.; Falenty, A.; Hansen, T. C. Extent and Relevance of Stacking Disorder in "Ice Ic." *Proc. Natl. Acad. Sci. U. S. A.* **2012**, *109*, 21259–21264.

(8) Malkin, T. L.; Murray, B. J.; Salzmann, C. G.; Molinero, V.; Pickering, S. J.; Whale, T. F. Stacking Disorder in Ice I. *Phys. Chem. Chem. Phys.* **2015**, *17*, 60–76.

(9) Hansen, T. C.; Koza, M. M.; Kuhs, W. F. Formation and Annealing of Cubic Ice: I. Modelling of Stacking Faults. *J. Phys. Condens. Matter* **2008**, *20*, 285104.

(10) Hansen, T. C.; Koza, M. M.; Lindner, P.; Kuhs, W. F. Formation and Annealing of Cubic Ice: II. Kinetic Study. *J. Phys. Condens. Matter* **2008**, *20*, 285105.

(11) Malkin, T. L.; Murray, B. J.; Brukhno, A. V.; Anwar, J.; Salzmann, C. G. Structure of Ice Crystallized from Supercooled Water. *Proc. Natl. Acad. Sci. U. S. A.* **2012**, *109*, 1041–1045.

(12) Playford, H. Y.; Whale, T. F.; Murray, B.; Tucker, M. G.; Salzmann, C. G. Analysis of Stacking Disorder in Ice I Using Pair Distribution Functions. *J. Appl. Crystallogr.* **2018**, *51*, 1211–1220.

AUTHOR INFORMATION

* Corresponding author: katrin.amannwinkel@fysik.su.se; amannk@mpip-mainz.mpg.de

[#]current address: Max Planck Institute for Polymer Research, 55128 Mainz & Institute of Physics, Johannes Gutenberg University Mainz, 55128 Mainz, Germany
